# Book Reviews

**Published:** 1914-11

**Authors:** 


### BOOK REVIEWS

ABDOMINAL OPERATIONS. By Sir Berkeley Moynihan, M. S., (London) F. R. C. S., Leeds, England. Third edition, entirely reset and enlarged. Two octavo volumes, totaling 980 pages, with 371 illustrations, 5 in colors. Philadelphia and London: W. B. Saunders Company, 1914. Cloth, $10 net; half Morocco, $13 net.

This splendid work on Abdominal Operations is undoubtedly the best book of its kind in the English language. The operations described are those in general use, and all, or almost all, of them are those practiced by Moynihan himself. The stamp of approval thus given them makes it of additional value as the reader is not confused by a rehash of various methods of operation. The illustrations are nearly all original and are drawn by Miss Ethel Wright, who has emphasized the essential features as needed.

---

CHEMISTRY AND TOXICOLOGY FOR NURSES. By Philip Asher, Ph. G., M. D., Dean and Professor of Chemistry at the New Orleans College of Pharmacy. 12 mo. of 190 pages. Philadelphia and London: W. B. Saunders Company, 1914. Cloth, $1.25 net.

This elementary discussion of chemistry is exceptionally well adapted to the use of nurses. The author apologizes in the preface for the elementary nature of the work. This, in reality, is essential value. When medical students are taught more from elementary books, instead of large text-books, they will have a clearer conception of medicine.

---

A LABORATORY MANUAL OF QUALITATIVE CHEMICAL ANALYSIS. By A. R. Bliss, Jr., M. D., Ph. G., Professor of Chemistry and Pharmacy in the Birmingham Medical College. Octavo of 244 pages, with working tables. Philadelphia and London: W. B. Saunders Company, 1914. Cloth, $2.00 net.

